# Clinical and Metabolic Predictors of Abnormal Uterine Bleeding in Women With Type 2 Diabetes Mellitus: A Retrospective Cohort Study

**DOI:** 10.7759/cureus.116527

**Published:** 2026-09-19

**Authors:** Ayisha Ejaz Bhutta, Zubia Bugti, Rizwana Naz, Mamoona Shuja, Muhammad Shahid Nawaz Khan, Shoaib Liaquat, Amina Shahid

**Affiliations:** 1 Obstetrics and Gynaecology, Kettering General Hospital, Kettering, GBR; 2 Obstetrics and Gynaecology, Sandeman Provincial Hospital, Quetta, PAK; 3 Obstetrics and Gynaecology, Unit One, Bolan University of Medical and Health Sciences, Quetta, PAK; 4 Internal Medicine, Al-Aleem Medical College, Lahore, PAK; 5 Medicine, Tertiary Care Hospital Nishtar-II, Multan, PAK; 6 Medicine, Nishtar Medical College and Hospital, Multan, PAK; 7 Research, Lahore University of Biological and Applied Sciences, Lahore, PAK

**Keywords:** abnormal uterine bleeding, body mass index, glycemic control, hba1c, metabolic dysfunction, obesity, type 2 diabetes mellitus

## Abstract

Background: Abnormal uterine bleeding (AUB) may be associated with metabolic and endocrine dysfunction in women with type 2 diabetes mellitus (T2DM), but factors associated with newly documented AUB in this population remain incompletely characterized.

Objective: To evaluate clinical and metabolic factors associated with incident AUB among premenopausal women with T2DM.

Methods: This multicenter retrospective cohort study included 150 women aged 18-55 years with established T2DM and no documented AUB at baseline. Clinical, anthropometric, and biochemical variables were obtained from records between January 2025 and January 2026. Incident AUB was defined according to the International Federation of Gynecology and Obstetrics criteria. Cox proportional hazards regression was used to estimate adjusted hazard ratios (aHRs) and 95% confidence intervals (CIs), with study center included in the multivariable model.

Results: During a median follow-up of 9.2 months, 45 women (30.0%) developed AUB. Women with incident AUB had higher HbA1c (8.8% vs 7.9%; p < 0.001), higher BMI (31.7 vs 28.9 kg/m²; p = 0.002), and longer diabetes duration (9.3 vs 7.1 years; p = 0.010). In multivariable analysis, HbA1c (aHR: 1.48 per 1% increase, 95% CI: 1.19-1.84), BMI (aHR: 1.41 per 5 kg/m² increase, 95% CI: 1.07-1.86), diabetes duration (aHR: 1.29 per five years, 95% CI: 1.01-1.65), and documented PCOS (aHR: 1.92, 95% CI: 1.04-3.55) were independently associated with incident AUB. Women with concurrent obesity and HbA1c ≥8.5% had the highest observed adjusted hazard (aHR: 3.68, 95% CI: 1.65-8.23).

Conclusion: Higher HbA1c and greater adiposity were consistently associated with incident AUB among women with T2DM. Routine assessment of menstrual symptoms may help identify women who warrant timely gynecological evaluation. These observational findings require confirmation in larger prospective studies.

## Introduction

Abnormal uterine bleeding (AUB) refers to disturbances in the frequency, regularity, duration, or volume of uterine bleeding. It is a major cause of anaemia, decreased quality of life, and health care utilization among women of reproductive and perimenopausal age [1-3]. The International Federation of Gynecology and Obstetrics (FIGO) PALM-COEIN (Polyp, Adenomyosis, Leiomyoma, Malignancy and hyperplasia, Coagulopathy, Ovulatory dysfunction, Endometrial, Iatrogenic, and Not otherwise classified) classification provides a standardized framework for distinguishing structural from nonstructural causes of AUB and has improved consistency in clinical evaluation and research reporting [4].

Abnormal menstrual and endometrial physiology may be associated with metabolic dysfunction. Insulin resistance and hyperinsulinemia may affect ovarian steroidogenesis, sex hormone-binding globulin levels, follicular growth, and luteal function, which in turn may lead to chronic or irregular exposure of the endometrium to estrogen without sufficient progesterone [5,6]. Emerging clinical evidence has also demonstrated associations between hyperinsulinemia, ovulatory dysfunction, and AUB, supporting a possible relationship between systemic metabolic status and endometrial physiology [7,8].

Such a relationship might be more significant in women with type 2 diabetes mellitus (T2DM), where chronic hyperglycemia is often accompanied by insulin resistance, obesity, dyslipidaemia, and low-grade inflammation [9]. Obesity could also affect reproductive physiology by altering peripheral estrogen production, adipokine signaling, inflammation, and ovulatory dysfunction. Polycystic ovary syndrome (PCOS), which is characterized by reproductive dysfunction and is frequently associated with insulin resistance and hyperandrogenism, represents an additional link between metabolic abnormalities and irregular menstrual function [10-13]. Experimental evidence also suggests that metabolic dysfunction and T2DM may influence endometrial proliferative pathways, providing further biological plausibility for an association between metabolic abnormalities and uterine health [14,15].

The relationship may also be bidirectional. Menstrual disturbances have been associated with adverse cardiometabolic profiles and an increased subsequent risk of metabolic disease, while insulin resistance and other metabolic abnormalities may contribute to menstrual dysfunction [11,12]. However, previous studies have largely focused on structural causes of AUB, reproductive dysfunction associated with obesity or PCOS, or menstrual abnormalities as predictors of future cardiometabolic disease. Comparatively less is known about the association between routinely measured metabolic characteristics and newly documented AUB among women with established T2DM [16,17].

This evidence gap is clinically relevant because glycated hemoglobin (HbA1c), body mass index (BMI), diabetes duration, and lipid parameters are routinely assessed during diabetes care and may help identify women who warrant closer assessment of menstrual symptoms [18]. The primary objective of this retrospective cohort study was to determine whether baseline clinical and metabolic characteristics, particularly HbA1c, BMI, diabetes duration, lipid profile, and PCOS, were independently associated with incident AUB among premenopausal women with T2DM. Secondary objectives were to describe the clinical phenotypes and structural findings of incident AUB and to explore the joint association of obesity and poor glycemic control with AUB occurrence. We hypothesized that higher HbA1c, greater adiposity, longer diabetes duration, and a less favorable metabolic profile would be associated with a higher hazard of incident AUB.

## Materials and methods

Study design and setting

This multicenter retrospective cohort study was conducted using clinical records from Kettering General Hospital, Kettering, United Kingdom, and Sandeman Provincial Hospital, Quetta, Pakistan, with research coordination through Lahore University of Biological and Applied Sciences (Lahore-UBAS), Lahore, Pakistan. Pre-menopausal women with T2DM with no AUB documented at baseline, followed up for newly documented AUB between January 2025 and January 2026, were included in the study population.

To minimise selection bias, potentially eligible records were screened chronologically, and consecutive records that fulfilled the predefined eligibility criteria were included. Finally, 150 women formed the analytical group. The total number of screening logs and individual numbers of records excluded for specific reasons could not be reliably reconstructed, as a complete screening log was not kept in the documentation of the available retrospective studies. There is a possibility of selection bias, and generalizability was taken into consideration in interpreting the findings. The study was reported using the Strengthening the Reporting of Observational Studies in Epidemiology (STROBE) guideline.

Study population

Women aged 18-55 years with documented T2DM were eligible if they had no documented AUB in the six months prior to the index date, had a baseline HbA1c and available metabolic data, and had at least one subsequent clinical follow-up after the index date. Women were excluded because they were either pregnant or within six months postpartum, were postmenopausal, had undergone hysterectomy, bilateral oophorectomy, or endometrial ablation or had a known gynecologic malignancy. Other exclusion criteria were as follows: symptomatic fibroids, adenomyosis, endometrial polyps, coagulation disorders, severe thrombocytopenia, end-stage kidney disease, decompensated liver disease, uncontrolled thyroid function, or baseline hyperprolactinemia.

Women receiving therapeutic anticoagulation, tamoxifen, menopausal hormone therapy, hormonal contraception, depot medroxyprogesterone acetate, or a levonorgestrel-releasing intrauterine system at baseline were also excluded. Records were excluded when baseline HbA1c was unavailable, menstrual status could not be established reliably, or follow-up was inadequate for outcome ascertainment.

PCOS status was abstracted from the available clinical records and treated as a pre-existing documented clinical diagnosis. Because this was a retrospective record-based study, the individual diagnostic components originally used to establish PCOS were not uniformly available for independent retrospective verification. PCOS was therefore analyzed as a documented clinical comorbidity rather than re-diagnosed according to a study-specific research protocol. This limitation was considered when interpreting associations involving PCOS.

Baseline clinical and metabolic assessment

The index date was defined as the first eligible clinical encounter during the study period for which sufficient clinical and biochemical information was available. Baseline measurements were obtained before any qualifying AUB event and as close as possible to the index date, within a maximum window of 90 days.

Recorded clinical variables included age, parity, body mass index (BMI), duration of T2DM, hypertension, dyslipidemia, PCOS, diabetic complications, smoking status, and glucose-lowering therapy. BMI was analyzed as a continuous variable, and obesity was defined as BMI ≥30 kg/m².

Metabolic measurements included HbA1c, fasting plasma glucose, total cholesterol, low-density lipoprotein cholesterol (LDL-C), high-density lipoprotein cholesterol (HDL-C), and triglycerides. HbA1c was the principal measure of glycemic exposure and was modeled per 1-percentage-point increase. Poor glycemic control was considered as HbA1c ≥8.5% for categorical analyses. Laboratory results were collected from institutional labs that were accredited and later transferred to common units prior to pooling.

Definition and ascertainment of abnormal uterine bleeding

The primary outcome was newly documented AUB after cohort entry. AUB was established using current FIGO guidelines, which include abnormalities in menstrual frequency, regularity, duration, or volume. Qualifying presentations were menstrual cycles with periods less than 24 days, greater than 38 days, clinically significant menstrual irregularity, bleeding for more than eight days, heavy bleeding demanding clinical assessment, and bleeding between periods. AUB was classified according to the predominant documented phenotype as heavy menstrual bleeding, irregular or frequent bleeding, prolonged menstrual bleeding, or intermenstrual bleeding. The date of the first qualifying episode was considered the event date. Potential AUB events were identified from gynecology, endocrinology, internal medicine, emergency department, imaging, hysteroscopy, endometrial sampling, and discharge records. Structural and nonstructural causes were interpreted according to the FIGO PALM-COEIN (Polyp, Adenomyosis, Leiomyoma, Malignancy and hyperplasia, Coagulopathy, Ovulatory dysfunction, Endometrial, Iatrogenic, and Not otherwise classified) framework. The same predefined outcome criteria were applied across both participating centers.

Only episodes supported by clinical documentation satisfying the predefined AUB criteria were considered qualifying events. Records in which menstrual status could not be established reliably were excluded during cohort assembly. Because outcome ascertainment was retrospective, formal prospective adjudication of every potential event was not possible. A subsample of records was independently cross-checked for eligibility and outcome classification as a data-quality measure. Potential under-documentation, differential detection, and outcome misclassification were therefore considered when interpreting the findings.

Women in whom structural uterine pathology was identified after the onset of AUB remained in the primary analysis because the principal outcome was incident AUB irrespective of subsequently established etiology. These women were excluded in a prespecified sensitivity analysis to determine whether the principal metabolic associations persisted after removal of structural causes.

Follow-up and data quality

Participants were monitored from the index date until the first documented AUB event, menopause, hysterectomy, death, loss to follow-up, or last available clinical follow-up, whichever came first. At both participating centers, the data-extraction framework, the variable definitions, the outcome criteria, and the coding rules were the same. Duplicate entries and implausible values were identified and verified against available source documentation, and analytical records were de-identified prior to analysis. To add consistency to retrospective data extraction, a subsample of records was cross-checked for eligibility and outcome classification, independently.

Potential center-related variations in referral patterns, clinical documentation, and diagnostic practice were taken into account in the analysis and interpretation of the data since the participating hospitals were in distinct health care systems.

Study outcomes

The primary endpoint was incident AUB during follow-up. The principal prespecified exposures of interest were HbA1c, BMI, diabetes duration, age, PCOS, triglycerides, and HDL-C. The primary analysis examined the independent associations between these baseline clinical and metabolic characteristics and time to incident AUB. Secondary analyses described the clinical phenotype and structural findings among women who developed AUB and explored the joint association of obesity and poor glycemic control with incident AUB.

For the joint metabolic analysis, participants were categorized into four predefined groups: BMI <30 kg/m² with HbA1c <8.5%, BMI ≥30 kg/m² with HbA1c <8.5%, BMI <30 kg/m² with HbA1c ≥8.5%, and BMI ≥30 kg/m² with HbA1c ≥8.5%. This analysis was intended to evaluate the observed accumulation of metabolic risk across categories and was not interpreted as evidence of biological interaction unless supported by a statistically significant interaction term.

Statistical analysis

Continuous variables were summarized as mean±standard deviation (SD) when approximately normally distributed or as median and interquartile range (IQR) when distributions were skewed. Categorical variables were reported as frequencies and percentages. Between-group comparisons of continuous variables were performed using the independent-samples Student's t-test or Mann-Whitney U test, as appropriate, with the corresponding t statistic or standardized Z statistic reported. Categorical variables were compared using Pearson's chi-square test, with the χ² statistic and corresponding P value reported.

Time-to-incident AUB was analyzed using Cox proportional-hazards regression, with hazard ratios (HRs), adjusted hazard ratios (aHRs), and 95% confidence intervals (CIs) estimated. HbA1c was modeled per 1-percentage-point increase, BMI per 5-kg/m² increase, and diabetes duration per five-year increase. The core multivariable model included age, HbA1c, BMI, diabetes duration, PCOS, and study center. Study center was incorporated into the core model to account for potential systematic differences between the two participating institutions. A prespecified metabolic extension additionally evaluated triglycerides and HDL-C.

Because only 45 incident AUB events occurred, model complexity was considered carefully in relation to the number of outcome events. Estimates from the expanded metabolic model were therefore interpreted cautiously because of the potential for model overfitting, reduced precision, and instability of estimates with wider CIs. The proportional-hazards assumption was evaluated using Schoenfeld residuals. Multicollinearity was assessed using correlation matrices and variance inflation factors. Model discrimination was evaluated using Harrell's C-index. Internal stability and optimism in model discrimination were assessed using 1,000 bootstrap resamples.

The joint association of obesity and poor glycemic control was evaluated using the four predefined BMI/HbA1c categories. A BMI-by-HbA1c interaction term was examined separately. A nonsignificant interaction term was interpreted as an absence of statistical evidence for multiplicative interaction and not as evidence of biological synergy. The specific statistical software version used for the original analysis could not be reliably reconstructed from the retained retrospective analytical documentation. Accordingly, the statistical procedures, model specifications, diagnostic assessments, and sensitivity analyses are described in detail to maximize methodological transparency. All statistical tests were two-sided, and p < 0.05 was considered statistically significant.

Missing data and sensitivity analyses

Records lacking baseline HbA1c, reliable menstrual status, or adequate follow-up were excluded before construction of the final analytical cohort. For individual analytical covariates, complete-case analysis was used when missingness was less than 5%. When covariate missingness exceeded 5%, multiple imputation by chained equations was used, with pooled estimates obtained according to Rubin's rules. The exact number of imputations and complete specification of the original imputation model could not be reliably reconstructed from the retained retrospective analytical documentation. This limitation was explicitly acknowledged because it reduces complete reproducibility of the missing-data procedure.

Prespecified sensitivity analyses were conducted to assess the robustness of the principal findings. These included exclusion of women in whom structural uterine pathology was identified after the onset of AUB; restriction of outcome ascertainment to AUB documented directly by gynecology services; additional adjustment for hypertension and insulin treatment; and categorical modeling of HbA1c and BMI using thresholds of ≥8.5% and ≥30 kg/m², respectively. These analyses were used to examine whether the principal associations were sensitive to structural causes of bleeding, a more restrictive definition of outcome ascertainment, additional cardiometabolic adjustment, or alternative specification of the principal exposures. All sensitivity analyses used two-sided testing, with p < 0.05 considered statistically significant.

Ethical considerations

Ethical approval for this retrospective clinical-record study was obtained from the Ethical Review Committee of Lahore University of Biological and Applied Sciences (Lahore-UBAS) under approval number ERC/2024/149A. All study data were anonymized before analysis, and confidentiality was maintained throughout the study. The investigation was conducted in accordance with the Declaration of Helsinki and applicable institutional and data-protection requirements. No study-specific intervention was performed, and the analysis was restricted to information available in existing clinical records.

## Results

Cohort characteristics and incidence of AUB

The analytical cohort comprised 150 women with T2DM. The mean age was 43.3±6.6 years, and the mean body mass index (BMI) was 29.7±4.9 kg/m². The median follow-up duration was 9.2 months (IQR: 8.0-10.5). During follow-up, 45 women developed incident AUB, corresponding to 30.0% of the analytical cohort.

Women who developed AUB had higher baseline HbA1c than women who remained AUB-free (8.8±1.3% vs 7.9±1.2%; t = 3.97, p < 0.001), corresponding to a mean difference of 0.9 percentage points (95% CI: 0.46-1.34). BMI was also higher among women who subsequently developed AUB (31.7±4.9 vs 28.9±4.7 kg/m²; t = 3.25, p = 0.002), and diabetes duration was longer (9.3±4.8 vs 7.1±4.4 years; t = 2.64, p = 0.010). Obesity and HbA1c ≥8.5% were more frequent among women who developed AUB. Fasting plasma glucose and triglyceride concentrations were higher, whereas HDL-C concentrations were lower. No statistically significant between-group differences were observed for age, total cholesterol, LDL-C, hypertension, insulin treatment, or metformin treatment (Table 1).

**Table 1:** Baseline characteristics according to incident abnormal uterine bleeding (AUB) Values are presented as mean±SD, median (IQR), or n (%), as appropriate. t=independent-samples t-test statistic; χ²=Pearson chi-square statistic; Z=standardized Mann-Whitney test statistic; AUB=abnormal uterine bleeding; BMI=body mass index; HbA1c=glycated hemoglobin; LDL-C=low-density lipoprotein cholesterol; HDL-C=high-density lipoprotein cholesterol; PCOS=polycystic ovary syndrome

Characteristic	AUB (n=45)	No AUB (n=105)	Test statistic	P value
Age, years	44.6±6.3	42.8±6.7	t=1.57	0.119
BMI, kg/m²	31.7±4.9	28.9±4.7	t=3.25	0.002
BMI ≥30 kg/m²	27 (60.0%)	42 (40.0%)	χ²=5.07	0.024
Diabetes duration, years	9.3±4.8	7.1±4.4	t=2.64	0.010
HbA1c, %	8.8±1.3	7.9±1.2	t=3.97	<0.001
HbA1c ≥8.5%	29 (64.4%)	38 (36.2%)	χ²=10.17	0.001
Fasting glucose, mg/dL	181±42	159±37	t=3.04	0.003
Total cholesterol, mg/dL	201±39	189±37	t=1.79	0.075
LDL-C, mg/dL	122±33	116±31	t=1.04	0.302
HDL-C, mg/dL	41.3±8.1	45.2±8.6	t=−2.65	0.009
Triglycerides, mg/dL	194 (158-238)	164 (132-203)	Z=2.88	0.004
Hypertension	21 (46.7%)	36 (34.3%)	χ²=2.05	0.152
PCOS	10 (22.2%)	11 (10.5%)	χ²=3.61	0.057
Insulin treatment	17 (37.8%)	28 (26.7%)	χ²=1.85	0.174
Metformin treatment	35 (77.8%)	87 (82.9%)	χ²=0.54	0.464

Clinical pattern of incident AUB

Heavy menstrual bleeding was the most frequently documented AUB phenotype, occurring in 21 of 45 women (46.7%). Irregular or frequent bleeding occurred in 12 women (26.7%), prolonged menstrual bleeding in seven (15.6%), and intermenstrual bleeding in five (11.1%). The median interval from cohort entry to the first documented AUB episode was 6.1 months (IQR: 4.2-8.3).

Structural uterine pathology was subsequently identified in nine women (20.0%) with incident AUB. Leiomyoma was the most frequent structural finding, followed by endometrial polyps, endometrial hyperplasia, and adenomyosis. No structural PALM abnormality was documented in 36 women (80.0%). No endometrial or cervical malignancy was documented during follow-up (Table 2).

**Table 2:** Clinical phenotype and structural findings among women with incident abnormal uterine bleeding (AUB) Values are presented as n (%). AUB=abnormal uterine bleeding

Characteristic	n (%)
Incident AUB	45 (100%)
Heavy menstrual bleeding	21 (46.7%)
Irregular/frequent bleeding	12 (26.7%)
Prolonged menstrual bleeding	7 (15.6%)
Intermenstrual bleeding	5 (11.1%)
Structural abnormality	9 (20.0%)
Leiomyoma	4 (8.9%)
Endometrial polyp	2 (4.4%)
Endometrial hyperplasia	2 (4.4%)
Adenomyosis	1 (2.2%)
No structural abnormality	36 (80.0%)

Associations with incident AUB

In univariable Cox proportional-hazards analyses, higher HbA1c, higher BMI, longer diabetes duration, documented PCOS, higher triglycerides, and lower HDL-C were associated with incident AUB. Each 1-percentage-point increase in HbA1c was associated with a 56% higher unadjusted hazard of AUB (HR: 1.56, 95% CI: 1.29-1.89; Wald χ² = 20.79, p < 0.001).

In the core multivariable model adjusting for age, HbA1c, BMI, diabetes duration, PCOS, and study center, HbA1c remained associated with incident AUB (aHR: 1.48 per 1-percentage-point increase, 95% CI: 1.19-1.84; Wald χ² = 12.46, p < 0.001). Each 5-kg/m² increase in BMI was associated with a 41% higher adjusted hazard (aHR: 1.41, 95% CI: 1.07-1.86; Wald χ² = 5.92, p = 0.015), and each five-year increase in diabetes duration was associated with a 29% higher adjusted hazard (aHR: 1.29, 95% CI: 1.01-1.65; Wald χ² = 4.18, p = 0.041). Documented PCOS was also associated with incident AUB (aHR: 1.92, 95% CI: 1.04-3.55; Wald χ² = 4.31, p = 0.038). Age was not independently associated with the outcome (aHR: 1.18 per five years, 95% CI: 0.94-1.49; Wald χ² = 1.86, p = 0.173).

In the prespecified metabolic extension of the model, triglycerides were associated with incident AUB (aHR: 1.17 per 50-mg/dL increase, 95% CI: 1.01-1.36; Wald χ² = 4.26, p = 0.039), while each 5-mg/dL decrease in HDL-C was associated with a 13% higher adjusted hazard (aHR: 1.13, 95% CI: 1.01-1.27; Wald χ² = 4.10, p = 0.043). Addition of the lipid variables resulted in only limited change in the HbA1c and BMI estimates. Because only 45 AUB events were observed, estimates from the expanded model should be interpreted cautiously because of the number of covariates relative to the number of outcome events (Table 3).

**Table 3:** Cox proportional-hazards analysis of factors associated with incident abnormal uterine bleeding (AUB) Adjusted estimates for the core variables were derived from the model including age, HbA1c, BMI, diabetes duration, PCOS, and study center. Triglyceride and HDL-C estimates were derived from the metabolic extension of the core model. Because the number of incident AUB events was limited, estimates from the expanded model should be interpreted with appropriate caution. HR=hazard ratio; CI=confidence interval; AUB=abnormal uterine bleeding; HbA1c=glycated hemoglobin; BMI=body mass index; PCOS=polycystic ovary syndrome; HDL-C=high-density lipoprotein cholesterol

Predictor	Unadjusted HR (95% CI)	Wald χ²	P value	Adjusted HR (95% CI)	Wald χ²	P value
HbA1c, per 1% increase	1.56 (1.29-1.89)	20.79	<0.001	1.48 (1.19-1.84)	12.46	<0.001
BMI, per 5 kg/m² increase	1.50 (1.16-1.94)	9.55	0.002	1.41 (1.07-1.86)	5.92	0.015
Diabetes duration, per 5 years	1.38 (1.10-1.74)	7.55	0.006	1.29 (1.01-1.65)	4.18	0.041
PCOS	2.12 (1.10-4.07)	5.02	0.025	1.92 (1.04-3.55)	4.31	0.038
Age, per 5 years	1.23 (1.00-1.51)	3.78	0.052	1.18 (0.94-1.49)	1.86	0.173
Triglycerides, per 50 mg/dL	1.22 (1.07-1.39)	8.81	0.003	1.17 (1.01-1.36)	4.26	0.039
HDL-C, per 5 mg/dL decrease	1.17 (1.05-1.30)	7.88	0.005	1.13 (1.01-1.27)	4.10	0.043

The proportional-hazards assumption was satisfied for the global model and individual covariates, and no important multicollinearity was identified. The core model yielded a Harrell C-index of 0.78 (95% CI: 0.71-0.85). After 1,000 bootstrap resamples, the optimism-corrected C-index was 0.75. These analyses suggested limited optimism in model discrimination; however, the relatively small number of outcome events remained an important consideration when interpreting model stability.

Joint association of obesity and poor glycemic control with incident AUB

The proportion of women developing AUB increased across the predefined joint categories of adiposity and glycemic control. AUB occurred in eight of 55 women (14.5%) with BMI <30 kg/m² and HbA1c <8.5%, eight of 28 women (28.6%) with obesity alone, nine of 26 women (34.6%) with HbA1c ≥8.5% alone, and 20 of 41 women (48.8%) with both obesity and HbA1c ≥8.5%.

Compared with women with neither metabolic abnormality, women with concurrent obesity and HbA1c ≥8.5% had a higher adjusted hazard of incident AUB (aHR: 3.68, 95% CI: 1.65-8.23; Wald χ² = 9.55, p = 0.002). The estimates for obesity alone and HbA1c ≥8.5% alone were also above unity, but their confidence intervals included 1.0. The BMI-by-HbA1c interaction term was not statistically significant (Wald χ² = 1.21, p = 0.271). Therefore, the observed pattern was interpreted as a graded increase across the predefined metabolic categories rather than evidence of a multiplicative interaction between BMI and HbA1c (Table 4).

**Table 4:** Joint association of obesity and glycemic control with incident abnormal uterine bleeding (AUB) The joint-category analysis was exploratory. The BMI-by-HbA1c interaction was not statistically significant (P=0.271); therefore, these findings should not be interpreted as demonstrating multiplicative or biological interaction. HR=hazard ratio; CI=confidence interval; BMI=body mass index; HbA1c=glycated hemoglobin; AUB=abnormal uterine bleeding

Metabolic category	n/events	Adjusted HR (95% CI)	Wald χ²	P value
BMI <30 kg/m², HbA1c <8.5%	55/8	Reference	-	-
BMI ≥30 kg/m², HbA1c <8.5%	28/8	1.73 (0.71-4.23)	1.45	0.229
BMI <30 kg/m², HbA1c ≥8.5%	26/9	2.15 (0.91-5.10)	3.04	0.081
BMI ≥30 kg/m², HbA1c ≥8.5%	41/20	3.68 (1.65-8.23)	9.55	0.002

Sensitivity analyses

The principal associations were broadly consistent across the prespecified sensitivity analyses. After exclusion of the nine women in whom structural uterine pathology was identified, HbA1c remained associated with incident AUB (aHR: 1.43 per 1-percentage-point increase, 95% CI: 1.13-1.82; Wald χ² = 8.81, p = 0.003), as did BMI (aHR: 1.38 per 5-kg/m² increase, 95% CI: 1.03-1.85; Wald χ² = 4.60, p = 0.032). The estimate for diabetes duration remained in the same direction but was less precise and no longer statistically significant (aHR 1.25 per five years, 95% CI: 0.96-1.63; Wald χ² = 2.72, p = 0.099).

When outcome ascertainment was restricted to AUB documented directly by gynecology services, the estimates remained similar for HbA1c (aHR: 1.45, 95% CI: 1.15-1.83; Wald χ² = 9.55, p = 0.002) and BMI (aHR: 1.39, 95% CI: 1.04-1.86; Wald χ² = 4.89, p = 0.027). Additional adjustment for hypertension and insulin treatment also resulted in limited changes in the estimates for HbA1c (aHR: 1.46, 95% CI: 1.17-1.82; Wald χ² = 10.83, p = 0.001) and BMI (aHR: 1.40, 95% CI: 1.06-1.85; Wald χ² = 5.60, p = 0.018).

When HbA1c and BMI were entered categorically rather than continuously, HbA1c ≥8.5% was associated with incident AUB (aHR: 2.14, 95% CI: 1.16-3.95; Wald χ² = 5.92, p = 0.015), as was BMI ≥30 kg/m² (aHR: 1.82, 95% CI: 1.01-3.29; Wald χ² = 3.95, p = 0.047). Overall, the direction and magnitude of the main HbA1c and BMI associations were generally consistent across alternative outcome definitions, exclusion of structural pathology, additional covariate adjustment, and categorical exposure specifications (Table 5).

**Table 5:** Sensitivity analyses of the principal associations with incident abnormal uterine bleeding (AUB) HR=hazard ratio; CI=confidence interval; AUB=abnormal uterine bleeding; HbA1c=glycated hemoglobin; BMI=body mass index

Sensitivity analysis	Adjusted HR (95% CI)	Wald χ²	P value
Excluding structural AUB			
HbA1c, per 1% increase	1.43 (1.13-1.82)	8.81	0.003
BMI, per 5 kg/m² increase	1.38 (1.03-1.85)	4.60	0.032
Diabetes duration, per 5 years	1.25 (0.96-1.63)	2.72	0.099
Gynecology-documented AUB only			
HbA1c, per 1% increase	1.45 (1.15-1.83)	9.55	0.002
BMI, per 5 kg/m² increase	1.39 (1.04-1.86)	4.89	0.027
Additional covariate adjustment			
HbA1c, per 1% increase	1.46 (1.17-1.82)	10.83	0.001
BMI, per 5 kg/m² increase	1.40 (1.06-1.85)	5.60	0.018
Categorical exposure analysis			
HbA1c ≥8.5%	2.14 (1.16-3.95)	5.92	0.015
BMI ≥30 kg/m²	1.82 (1.01-3.29)	3.95	0.047

Overall, higher HbA1c and greater BMI showed the most consistent associations with incident AUB across the primary and sensitivity analyses. Longer diabetes duration, documented PCOS, higher triglycerides, and lower HDL-C were also associated with incident AUB in the fitted models, although these estimates should be interpreted in the context of the limited number of outcome events and the retrospective study design. The highest observed proportion of AUB occurred among women with concurrent obesity and HbA1c ≥8.5%; however, the absence of a statistically significant BMI-by-HbA1c interaction does not support a conclusion of multiplicative interaction or biological synergy.

## Discussion

In this multicenter retrospective cohort of premenopausal women with T2DM, 30.0% developed newly documented AUB during a median follow-up of 9.2 months. Higher HbA1c, greater BMI, longer diabetes duration, documented PCOS, higher triglycerides, and lower HDL-C were associated with incident AUB in the fitted models. HbA1c and BMI showed the most consistent associations across the primary and sensitivity analyses. These associations also persisted after women with subsequently identified structural uterine pathology were excluded, suggesting that metabolic characteristics may be relevant to AUB occurring without an identified structural PALM abnormality. Current FIGO frameworks recognize that nonstructural mechanisms may contribute substantially to AUB [1-4].

Heavy menstrual bleeding was the most frequently documented phenotype, while most affected women had no structural uterine abnormality identified during follow-up. Contemporary approaches to AUB emphasize that abnormal bleeding may arise from structural, ovulatory, endometrial, systemic, or iatrogenic mechanisms and should therefore be evaluated systematically rather than attributed to a single pathological process [1,4-7]. The present findings consequently do not replace conventional gynecological evaluation but suggest that metabolic status may represent an additional clinically relevant consideration in women with T2DM [4,7].

Poorer glycemic control showed a consistent association with incident AUB, with each 1-percentage-point increase in HbA1c associated with a 48% higher adjusted hazard. Insulin resistance and hyperinsulinemia may influence ovarian steroidogenesis, sex hormone-binding globulin concentrations, follicular development, and ovulation, potentially resulting in prolonged or irregular exposure of the endometrium to estrogen [8-10]. Clinical evidence has also demonstrated an association between hyperinsulinemia and AUB, providing biological plausibility for an association between metabolic dysfunction and abnormal endometrial shedding [11]. These mechanisms were not directly measured in the present study and should therefore be regarded as possible explanations rather than demonstrated causal pathways.

PCOS further illustrates the relationship between metabolic and reproductive dysfunction. Insulin resistance, hyperandrogenism, and chronic ovulatory dysfunction are important components of the metabolic and reproductive phenotype of PCOS [13-15]. Documented PCOS was associated with an approximately twofold higher adjusted hazard of AUB in this cohort, while HbA1c and BMI remained associated with AUB after adjustment for PCOS. This suggests that the observed metabolic associations were not confined to women with PCOS. Menstrual irregularities have also been associated with adverse cardiometabolic profiles and subsequent metabolic disease, supporting a potentially bidirectional relationship between reproductive and metabolic health [12-15].

Greater adiposity was also associated with incident AUB. Women with concurrent obesity and HbA1c ≥8.5% had the highest observed proportion of AUB events. Obesity may influence ovarian and endometrial physiology through peripheral estrogen production, altered adipokine signaling, insulin resistance, chronic inflammation, and ovulatory dysfunction [16,17]. Previous evidence also suggests that weight reduction may influence some endometrial abnormalities, further supporting a relationship between adiposity and uterine health [18]. However, the BMI-by-HbA1c interaction in the present study was not statistically significant; therefore, the increasing event proportions across the metabolic categories should be interpreted as a graded accumulation of observed risk rather than evidence of multiplicative interaction or biological synergy.

Longer diabetes duration, higher triglycerides, and lower HDL cholesterol were additionally associated with incident AUB in the fitted models. These characteristics may represent markers of prolonged metabolic dysfunction rather than direct causes of uterine bleeding. Experimental evidence suggests that T2DM may influence endometrial proliferative pathways, while insulin-sensitizing approaches have been investigated in endometrial disorders [19,20]. Such evidence provides biological context for the observed associations but does not establish that metabolic abnormalities directly caused AUB in the present cohort [19,20].

From a clinical perspective, routinely available measures, such as HbA1c, BMI, diabetes duration, and lipid profile, may help identify women in whom menstrual symptoms deserve closer attention. AUB can substantially affect quality of life and may arise from several structural and nonstructural mechanisms requiring systematic evaluation [1-4]. Metabolic characteristics should therefore be considered an additional component of clinical assessment rather than a substitute for appropriate gynecological investigation [4-7].

The study has several strengths. Baseline metabolic exposures were assessed before the first documented AUB event, predefined clinical and metabolic variables were evaluated, the study center was incorporated into the multivariable analysis, and proportional-hazards assumptions and multicollinearity were assessed. Furthermore, the principal associations of HbA1c and BMI remained broadly consistent after exclusion of structural AUB, restriction to gynecology-documented events, additional cardiometabolic adjustment, and alternative categorical exposure definitions. These sensitivity analyses increase confidence in the internal consistency of the findings, although they cannot eliminate limitations inherent to an observational study.

Several limitations should be considered. First, the retrospective design depended on the completeness and accuracy of routine clinical documentation. Mild or unreported menstrual disturbances may therefore have been missed, and women with more frequent healthcare contact may have had AUB identified more readily. Second, structural uterine abnormalities may have been under-recognized because uniform prospective imaging, hysteroscopy, or endometrial evaluation was not performed in every participant. Such limitations are particularly important in AUB because contemporary diagnostic frameworks emphasize systematic evaluation for both structural and nonstructural causes [4-7].

Confounding is also a possibility. There were also cases in which the reproductive, hormonal, gynecological, behavioral, and treatment-related variables were not consistently recorded and thus were not included in the multivariable analyses. PCOS was also derived from a prior clinical diagnosis, not by application of standardized research criteria, which was a potential source of exposure misclassification. This is pertinent as PCOS, insulin resistance, adiposity and ovulatory dysfunction are all closely interconnected [13-17].

The two participating hospitals were in different countries and in different healthcare systems. Center-level heterogeneity may thus be attributable to differences in referral patterns, population characteristics, clinical documentation, diagnostic practice, and access to gynecological assessment. Study center was included in the multivariable model, but statistical adjustment cannot fully adjust for unmeasured institutional or population differences. Thus, caution must be exercised when extrapolating the results to other populations and healthcare settings than those included in this cohort.

A significant restriction was the relatively small number of outcome events. Several covariates were assessed in the multivariable Cox models, yet only 45 women developed an incident AUB. While the core model was prespecified and bootstrap resampling was performed to evaluate the internal stability, the number of events in relation to the complexity of the model suggests potential overfitting and imprecision, especially for the expanded metabolic model. As a result, one should exercise caution when interpreting estimates with larger CIs or with borderline statistical significance.

However, the median follow-up (9.2 months) was relatively brief and may not have been sufficient to see AUB develop following longer metabolic exposure. More comprehensive evaluation of temporal association between metabolic dysfunction and AUB would require longer prospective observation with uniform menstrual assessment and repeated metabolic measurements. The literature also corroborates the significance of a comprehensive evaluation of menstrual and cardiometabolic health over time [4,12].

Future prospective multicenter studies should incorporate standardized AUB ascertainment, uniform gynecological assessment, predefined PCOS diagnostic criteria, comprehensive measurement of reproductive and hormonal confounders, and longer follow-up. Interventional studies would additionally be required to determine whether improving glycemic control, reducing adiposity, or modifying other metabolic abnormalities actually reduces subsequent AUB rather than simply being associated with a lower observed risk [12,18-20].

## Conclusions

Among premenopausal women with T2DM, higher HbA1c and greater BMI were consistently associated with newly documented AUB, while longer diabetes duration, documented PCOS, higher triglycerides, and lower HDL-C also showed associations in the fitted models. Women with concurrent obesity and HbA1c ≥8.5% had the highest observed proportion of AUB; however, the absence of a statistically significant BMI-by-HbA1c interaction does not support a conclusion of multiplicative interaction or biological synergy.

Routine assessment of menstrual symptoms during follow-up of women with T2DM may help identify individuals who require timely gynecological evaluation, particularly those with less favorable metabolic profiles. Nevertheless, the findings should be interpreted in the context of the retrospective design, limited number of outcome events, potential residual confounding, center-level heterogeneity, and relatively short follow-up. Larger prospective studies are required to confirm these associations and to determine whether modification of glycemic control, adiposity, or other metabolic factors can reduce the subsequent occurrence of AUB.
